# Retention and Transmission of Grapevine Leafroll-Associated Virus 3 by *Pseudococcus calceolariae*

**DOI:** 10.3389/fmicb.2021.663948

**Published:** 2021-05-12

**Authors:** Brogan McGreal, Manoharie Sandanayaka, Rebecca Gough, Roshni Rohra, Vicky Davis, Christina W. Marshall, Kate Richards, Vaughn A. Bell, Kar Mun Chooi, Robin M. MacDiarmid

**Affiliations:** ^1^The New Zealand Institute for Plant and Food Research Limited (PFR), Auckland, New Zealand; ^2^School of Biological Sciences, The University of Auckland, Auckland, New Zealand; ^3^The New Zealand Institute for Plant and Food Research Limited (PFR), Hastings, New Zealand

**Keywords:** *Pseudococcus calceolariae*, grapevine leafroll-associated virus 3, alternative host, retention, transmission, clover, *Trifolium repens*

## Abstract

Grapevine leafroll-associated virus 3 (GLRaV-3), an economically significant pathogen of grapevines, is transmitted by *Pseudococcus calceolariae*, a mealybug commonly found in New Zealand vineyards. To help inform alternative GLRaV-3 control strategies, this study evaluated the three-way interaction between the mealybug, its plant host and the virus. The retention and transmission of GLRaV-3 by *P. calceolariae* after access to non-*Vitis* host plants (and a non-GLRaV-3 host) White clover (*Trifolium repens* L. cv. “Grasslands Huia white clover”), Crimson clover (*T. incarnatum*), and *Nicotiana benthamiana* (an alternative GLRaV-3 host) was investigated. For all experiments, *P. calceolariae* first instars with a 4 or 6 days acquisition access period on GLRaV-3-positive grapevine leaves were used. GLRaV-3 was detected in mealybugs up to 16 days on non-*Vitis* plant hosts but not after 20 days. GLRaV-3 was retained by second instars (*n* = 8/45) and exuviae (molted skin, *n* = 6/6) following a 4 days acquisition period on infected grapevines leaves and an 11 days feeding on non-*Vitis* plant hosts. Furthermore, GLRaV-3 was transmitted to grapevine (40−60%) by *P. calceolariae* second instars after access to white clover for up to 11 days; 90% transmission to grapevine was achieved when no alternative host feeding was provided. The 16 days retention period is the longest observed in mealybug vectoring of GLRaV-3. The results suggest that an alternative strategy of using ground-cover plants as a disrupter of virus transmission may be effective if mealybugs settle and continue to feed on them for 20 or more days.

## Introduction

Grapevine leafroll-associated virus 3 (GLRaV-3) is the main and most widespread etiological agent of grapevine leafroll disease (GLD) worldwide (Maree et al., 2013). GLD negatively affects berry yield and qualitative characteristics like soluble solids, titratable acidity, and anthocyanins (Over de Linden and Chamberlain, 1970; Cabaleiro et al., 1999; Charles et al., 2006; Lee and Martin, 2009; Lee et al., 2009; Vega et al., 2011; Martelli, 2014; Montero et al., 2016). GLRaV-3 is transmitted by propagation and grafting of infected grapevine material and by insect vectors, namely mealybugs, soft scale and scale insects (Maree et al., 2013). It has never been demonstrated that GLRaV-3 is transmitted mechanically.

Mealybugs (Hemiptera: Pseudococcidae) are commonly found in New Zealand vineyards. Two cosmopolitan species are especially problematic because they transmit GLRaV-3: *Pseudococcus calceolariae* and *P. longispinus* (Charles, 1993).

*Pseudococcus calceolariae* and *P. longispinus* are both polyphagous, feeding on numerous plant species throughout New Zealand, including horticultural crops such as apple and pear, and ground-cover species such as white clover, red clover, and doves foot (Charles, 1993; Charles et al., 2006). New Zealand’s cool climate status means *P. calceolariae* and *P. longispinus* have two to three generations per year, but can reach a fourth generation under favorable warmer conditions (Charles et al., 2006). *Pseudococcus calceolariae* colonizes all parts of the grapevine, including the roots. Petersen and Charles (1997) demonstrated that *P. calceolariae* first instars transmit GLRaV-3 efficiently but little is known about the length of time required for virus to be acquired and retained by this vector species.

In New Zealand, mealybugs are the key contributor of GLRaV-3 spread from infected grapevines to adjacent healthy vines in vineyards (Bell et al., 2018). Consequently, active control of mealybug populations is recognized as an integral component of a successful GLRaV-3 management program (Bell et al., 2018). Knowledge of virus acquisition, retention, and transmission is fundamental to understanding the interaction between plant viruses and the insect vectors in the context of a range of mealybug plant hosts. Specifically, understanding transmission biology and the impact of non-*Vitis* food sources for mealybug could be important for the further development and enhancement of GLRaV-3 management responses (Almeida et al., 2013; Krüger et al., 2015).

Mealybug numbers are generally maintained at low population densities in New Zealand vineyards through spring applications of insecticides compatible with integrated pest management (IPM; e.g., buprofezin). This response helps facilitate biological control later in the growing season (Charles et al., 2010). Thus, IPM-compatible chemistry and biological control are believed to greatly minimize the risk of vector-mediated transmission of GLRaV-3 (Bell et al., 2018). In addition, grapevines identified as GLRaV-3 infected are rogued (removed) to reduce inoculum in target areas, with the missing vines replaced with those sourced from a nursery certified by the wine sector to supply healthy vines (Bell et al., 2018). However, within New Zealand’s Hawke’s Bay viticulture region, two organically managed (minimal application of pesticides) vineyards presented high initial GLD incidence but low GLRaV-3 transmission despite apparent high mealybug numbers observed on the ground cover plants (Bell et al., 2018). Notably, mealybug populations on grapevines in both vineyards were low (<3 mealybugs per 100 vine leaves inspected) for at least 6 years (Bell et al., 2018). In other words, there was no evidence of large-scale *P. calceolariae* migration from groundcover to grapevine in either vineyard, an observation supported by a substantially reduced influence of GLRaV-3 over time. In 2009, GLRaV-3 incidence was quantified at 10% in one organically managed vineyard planted in mature Merlot vines, and at 16% in the second planted in mature Cabernet Sauvignon. Once the initial infected vines in each vineyard were removed, annual incidence was consistently less than 1% from years 2 to 6, when monitoring concluded (Bell et al., 2018). Therefore, high mealybug numbers within vineyards may not always result in GLRaV-3 transmission to grapevine. This may be associated with mealybug feeding on ground-cover plants rather than grapevines.

Preliminary retention and transmission experiments were carried out in 2015, with retention experiments repeated in 2019–2020 (2020 Retention experiments). The aims of this study were to determine (i) the GLRaV-3 retention period in viruliferous first instar *P. calceolariae* after feeding on non-*Vitis* plant hosts, (ii) whether GLRaV-3 is retained in second instar *P. calceolariae* and exuviae molted by the first instar *P. calceolariae* after feeding on non-*Vitis* plant hosts, and (iii) whether GLRaV-3 is transmissible by viruliferous first and second instar *P. calceolariae* after feeding on non-*Vitis* plant hosts. White clover (*Trifolium repens* L. cv. Grasslands Huia white clover), Crimson clover (*Trifolium incarnatum*) and *Nicotiana benthamiana* were used as non-*Vitis* plant hosts, of which only *N. benthamiana* has been demonstrated as an alternative host for GLRaV-3.

## Materials and Methods

### Plant Material

For the retention and transmission experiments in 2015, GLRaV-3-positive leaf material was obtained from *V. vinifera* cv. Pinot noir and *V. vinifera* cv. Sauvignon blanc grapevines infected with GLRaV-3 group I maintained in controlled growth rooms (23°C with a 16 h light and 8 h dark cycle). For GLRaV-3-negative plant material, Cabernet franc were sourced from a New Zealand grapevine collection (Lincoln, New Zealand; New Zealand Winegrowers) and Merlot from Riversun Nursery (Gisborne, New Zealand). The virus status of all plants was confirmed by enzyme-linked immunosorbent assay (ELISA) or immunocapture RT-qPCR (Blouin et al., 2017). White clover (*Trifolium repens* L. cv. Grasslands Huia White clover) plants were grown from seed and were used as the non-*Vitis* host plant.

For 2020 retention experiments, GLRaV-3 was obtained from Pinot noir grapevines infected with at least two GLRaV-3 genetic variants representative of phylogenetic groups I and VI. White clover (*Trifolium repens* L. cv. Grasslands Huia white clover), Crimson clover (*Trifolium incarnatum*) and *Nicotiana benthamiana* plants were grown from seed and used as supplied to *P. calceolariae*. These the non-*Vitis* host plants were maintained within a glasshouse (at average 24°C with 16 h light and 8 h dark cycle) within separate, species-specific units with adequate space between plants. To ensure each donor leaf was infected with GLRaV-3 and sufficient GLRaV-3 was acquired by mealybugs, the petiole from each grapevine donor leaf was collected and tested by RT-qPCR (McGreal et al., 2019).

### *Pseudococcus calceolariae* Colonies

*Pseudococcus calceolariae* mealybugs were reared and maintained at The New Zealand Institute for Plant and Food Research (PFR), Auckland campus, within vented, plastic containers held under controlled laboratory conditions (22°C with 16 h light and 8 h dark cycle). The colonies were sustained on seed potatoes over multiple generations without exposure to grapevines or GLRaV-3.

### GLRaV-3 Retention by *Pseudococcus calceolariae*

Retention experiments were performed in both 2015 and 2020 as detailed below and summarized in Table 1.

**TABLE 1:** Summary of retention experiments performed in 2015 and 2020.

**Treatment name**	**Year (number of experimental replicates)**	**Acquisition access period (AAP) or mock (mealybug age)**	**Days post initial acquisition on grapevine or non-*Vitis* host (mealybug age)**
Virus	2015 (×2)	GLRaV-3 positive grapevine (1–4 days old)	Days 1–4 on original GLRaV-3 positive grapevine (5–8 days old^a,b^′)
Virus/White clover	2015 (×2)	GLRaV-3 positive grapevine (1–4 days old)	Days 1–4 on White clover (5–8 days old^a,b^)
Virus/Grapevine	2015 (×2)	GLRaV-3 positive grapevine (1–4 days old)	Days 1–4 on GLRaV-3 negative grapevine (5–8 days old^a^)
No Virus	2015 (×2)	GLRaV-3 negative grapevine (1–4 days old)	Days 1–4 on GLRaV-3 negative grapevine (5–8 days old^a,b^)
No Virus/White clover	2015 (×2)	GLRaV-3 negative grapevine (1–4 days old)	White clover (5–8 days old^a,b^)
Virus/White clover	2020 (×1)	GLRaV-3 positive grapevine (1–6 days old)	Days 1–40 on White clover (7–46 days old^c^)
Virus/Crimson clover	2020 (×1)	GLRaV-3 positive grapevine (1–6 days old)	Days 1–40 on Crimson clover (7–46 days old^c^′)
Virus/*Nicotiana benthamiana*	2020 (×1)	GLRaV-3 positive grapevine (1–6 days old)	Days 1–16 on *Nicotiana benthamiana* (7–22 days old^c^′′)

*^*a*^First instar mealybugs collected after 1, 2, 3, and 4 days after feeding on this plant host then tested for GLRaV-3.^*b*^Second instar and excuviae collected directly from this treatment then tested for GLRaV-3.^*b*^′Second instar and excuviae collected from this treatment following transfer of first instars at AAP 10–12 days to Petri dish, then tested for GLRaV-3.^*c*^First instar mealybugs collected after 5, 10, 15, 20, and 40 days after feeding on this plant host then tested for GLRaV-3.^*c*^′First instar mealybugs collected after 5, 10, 15, and 20 days after feeding on this plant host then tested for GLRaV-3, as the plants were sprayed at day 35 to treat a pest infestation.^*c*^′′First instar mealybugs collected after 5 and 10 days after feeding on this plant host then tested for GLRaV-3, as no mealybugs were present on the plant thereafter.*

#### 2015 Retention Experiment (Days 1–4 on Grapevine or a Non-*Vitis* Host)

*Pseudococcus calceolariae* eggs were collected from the colony and placed on a moist, black filter paper for emergence of first instars. Less than 24 h old nymphs were transferred to excised GLRaV-3-positive Cabernet franc grapevine leaves for an acquisition access period (AAP) or to GLRaV-3-free grapevine leaves for a mock AAP. Excised leaves were kept in good condition within vertically-orientated Petri dishes with the petiole extending through a fitted foam plug placed through the base of both lid and dish into a tube of water. After a 4 days AAP, *P. calceolariae* nymphs were transferred with a paint brush to White clover (Virus/White clover), GLRaV-3-free grapevine leaves (Virus/Grapevine), or maintained on the virus-infected leaves as a positive control (Virus). After a 4 days mock AAP, nymphs were transferred to White clover (No virus/White clover) or maintained on the uninfected grapevine leaves as a negative control (No virus). For all five treatments (Virus/White clover, No virus/White clover, Virus/Grapevine, Virus, and No virus), first instar *P. calceolariae* were collected after 1, 2, 3, and 4 days feeding on grapevine or the non-*Vitis* host. To minimize disruption to mealybug feeding they were collected from separate grapevine or White clover leaves each day. Second instar and exuviae were collected from four treatments (Virus/White clover, No virus/White clover, Virus, and No virus). For the Virus treatment, late first instar mealybugs close to molt (AAP 10–12 days) were transferred onto moist filter paper in a Petri dish to ensure that no nascent second instar mealybugs had an opportunity to feed on GLRaV-3-positive leaves. A maximum of 10 first instar mealybugs were transferred into each Petri dish, thereafter the plates were checked daily for exuviae and second instar mealybugs. The second instar mealybugs with recently removed exuviae were identified based on their shiny brown skin and the absence of the wooly appearance characteristic of first instars. For treatments Virus/White clover, No virus/White clover or No virus, second instar and exuviae were sampled directly from White clover or virus-free grapevine leaves, respectively. GLRaV-3 status in mealybugs and exuviae was detected by one-step RT-qPCR as described previously (McGreal et al., 2019). The GLRaV-3 retention assay was repeated in the same year.

#### 2020 Retention Experiment (Days1–40 on a Non-*Vitis* Host)

*Pseudococcus calceolariae* egg masses with emerging nymphs were added to an excised GLRaV-3 donor grapevine leaf (∼400 eggs per grapevine leaf). Each grapevine leaf was maintained in a Petri dish as described above. After 24 h the unhatched eggs were removed, leaving the newly emerged nymphs on the leaf surface (∼300 mealybug nymphs/leaf). After a 6 days AAP on virus donor grapevine leaves (two additional days to the 2015 experiment in an attempt to increase the percentage of viruliferous individuals) viruliferous mealybugs were transferred most delicately onto recipient plants by cutting donor grapevine leaf pieces harboring ∼40 mealybugs (per non-*Vitis* host plant) and placing the mealybug-loaded leaf piece(s) onto each non-*Vitis* host plant. The grapevine leaf pieces were removed once they were mealybug free. Mealybugs remained on each non-*Vitis* host for up to 40 days, with harvesting of subsets at 5, 10, 16, 20, and 40 days. Once the mealybug collection was completed, the remaining mealybugs were killed by spraying the plants with insecticide (a mixture of Movento^®^, Avid^®^, and Confidor^®^ with Partner^®^).

To test GLRaV-3 retention in mealybugs after feeding on clover or *N. benthamiana*, five mealybugs from each non-*Vitis* host plant per time point were collected, with individual mealybugs placed into Eppendorf tubes. Mealybug males in cocoons were also included in the sample collection. No mealybugs were present on *N. benthamiana* plants after 16 days. At 35 days post-inoculation, Crimson clover plants were treated with Confidor^®^ for an infestation of aphids, mites and whiteflies that killed 14 plants. After the final mealybug sample collection on day 40, all the plants were treated with a mixture of Movento^®^, Avid^®^, and Confidor^®^ with Partner^®^. At Day 0 (after 6 days AAP), three mealybugs from each donor leaf were collected into individual Eppendorf tubes. The individual donor petioles and the individual mealybug samples were tested for GLRaV-3 as described previously (McGreal et al., 2019). A negative mealybug control (not fed on GLRaV-3 infected host plant) and a buffer only control was included in each batch (10–22 mealybugs) of RNA extractions and RT-qPCR to ensure that no false positives were recorded within the 1,288 mealybug dataset. The GLRaV-3 status in mealybugs on the non-*Vitis* plant for 5, 10, 16, 20, and 40 days was assessed by RT-qPCR (McGreal et al., 2019).

### GLRaV-3 Transmission by *Pseudococcus calceolariae*

After a 4 days AAP on excised GLRaV-3-positive grapevine leaves, *P. calceolariae* nymphs were transferred to White clover leaves (as performed for the 2015 retention experiments). Following either a 5 or 11 days non-*Vitis* plant feeding on White clover, *P. calceolariae* nymphs were inoculated on GLRaV-3-free Merlot grapevine plants, as first (Treatment 1) or second instars (Treatment 2), respectively. As a positive control, *P. calceolariae* nymphs were maintained on GLRaV-3-positive leaves for a total of 10 or 16 days AAP, and thereafter transferred to GLRaV-3-free Merlot grapevine plants as either first (Treatment 3) or second instars (Treatment 4), respectively. For the inoculation of GLRaV-3-free grapevine plants, large healthy leaves, two or three nodes above the grapevine graft union, were selected. White clover or GLRaV-3 positive leaves with 25–50 mealybugs were secured to the underside of the grapevine leaf with Blu-Tack (Bostik). Mealybugs were maintained for a 7–10 days inoculation access period (IAP) on GLRaV-3-free grapevines, thereafter plants were sprayed with Movento^®^. Plants were physically separated during the IAP. Grapevines were pruned and maintained in the glasshouse for 5 months and were sprayed with insecticides once a month. Previous research by Cohen et al. (2004) showed that initial GLRaV-3 spread after transmission was basipetal from the graft point. Therefore, pruning of growth more than three nodes above the marked leaf (on which the mealybugs were inoculated), should have had a minimal effect on distribution of the transmitted GLRaV-3. The GLRaV-3 transmission experiment was performed twice in 2015. Four (Block 2) to five months (Block 1) after GLRaV-3 transmission onto previously GLRaV-3-negative grapevine plants, each inoculated leaf to which mealybugs had been transferred and basipetal cane sections were sampled and tested for GLRaV-3 as described previously (McGreal et al., 2019). As a negative control, GLRaV-3-free Merlot grapevine plants were maintained in the glasshouse until all plants were tested for GLRaV-3 (Prator et al., 2017).

### Statistical Analysis

To provide accuracy when expected values are small, Fisher’s Exact Test was used to analyze the proportion of GLRaV-3 positive mealybugs to the total number of mealybugs tested for the different feeding treatments and to compare the proportion of GLRaV-3 positive grapevine to the total number of grapevines. To enable comparison between treatments where mealybugs were fed on GLRaV-3 positive grapevine leaves (which was skewed when compared with negative controls where mealybugs were fed on GLRaV-3-free grapevine leaves), the 2015 retention dataset was blocked based on expected positive samples (Virus, Virus/White clover and Virus/Grapevine) and expected negative samples (No virus and No virus/White clover). The following variables were compared: GLRaV-3 status, days feeding on alternate host (grapevine or non-*Vitis*), treatment, source plant and alternate host. The false discovery rate correction (fdr) was applied when performing multiple *post-hoc* pairwise comparisons (Benjamini and Hochberg, 1995). Analyses were implemented in R version 3.6.1 (Team, 2015).

## Results

### GLRaV-3 Retention in *Pseudococcus calceolariae*

#### 2015 Retention Experiments: Days 1–4 on Grapevine or a Non-*Vitis* Host

**FIGURE 1:**
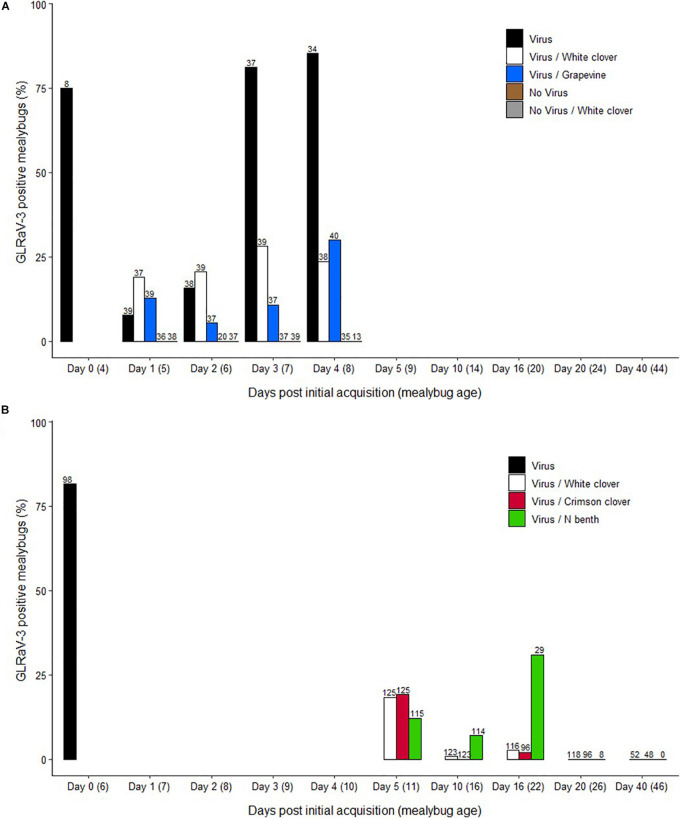
Retention of grapevine leafroll-associated virus 3 (GLRaV-3) in *Pseudococcus calceolariae* after feeding on non-*Vitis* plant host based on results from experiments conducted in **(A)** 2015 and **(B)** 2020. **(A)** Day 0 (Virus acquisition): First instar nymphs had a 4 days acquisition access period (AAP) on GLRaV-3 positive grapevine leaves, and a sub-sample were tested for GLRaV-3. Days 1–4: Nymphs were transferred to and allowed a 1–4 days access period on White clover (*Trifolium repens* L.) (Virus/White Clover) or GLRaV-3 negative grapevines (Virus/Grapevine). As a positive control, mealybugs were left on GLRaV-3 positive grapevine leaves for the duration of the experiment (Virus). Two negative controls were: mealybugs with a 4 days AAP on GLRaV-3 negative grapevine leaves, and then transferred on to White clover (No virus/White Clover) and mealybugs left on GLRaV-3 negative grapevine leaves for the duration of the experiment (No virus). Mealybugs were sampled each day and tested for GLRaV-3. **(B)** Day 0: First instar nymphs had a 6 days AAP on GLRaV-3 positive grapevine leaves and a sub-sample was tested for GLRaV-3 (Virus). Days 5–40: Nymphs were transferred to allow a 5–40 days access period on Crimson clover (*T. incarnatum*), White clover or *Nicotiana benthamiana*. Mealybugs were sampled from White clover (Virus/White clover), Crimson clover (Virus/Crimson clover), and *N. benthamiana* (Virus/N benth) in ∼5 days increments and tested for GLRaV-3. Sample numbers (n) are included above each bar.

Samples positive for GLRaV-3 generated Ct values that ranged between 24 and 38. The percentage of single, viruliferous mealybugs was 7, 15, 81, and 84% after 1, 2, 3, and 4 days post initial AAP, respectively (Figure 1A). Based on pairwise comparison using the Fisher exact test and fdr correction, there was evidence of a statistically significant difference between Day 1 (7%), and Day 3 (81%) and Day 4 (84%) (*p* < 0.001) and between Day 2 (15%) and both Days 3 and 4 (*p* < 0.001). When feeding on a non-*Vitis* host plant (Virus/White clover) or on GLRaV-3-free grapevine leaves (Virus/Grapevine), *P. calceolariae* nymphs retained GLRaV-3 for at least 4 days (Figure 1A). For the Virus/White clover treatment, the percentage of viruliferous mealybugs ranged over time from 18 to 31%. Over time for the Virus/Grapevine treatment, the percentage of viruliferous mealybugs ranged from 5 to 30%. For both Virus/White clover and Virus/Grapevine treatments, there was no evidence of statistically significant difference in the percentage of viruliferous mealybugs (*p* > 0.05). The percentages of viruliferous mealybugs varied for each of the GLRaV-3 retention treatments (Virus/White clover, Virus/Grapevine and Virus) on Days 3 and 4 post initial AAP (Figure 1A). At Day 3, there was evidence of a difference between the Virus (81%) and both Virus/White clover (31%) and Virus/Grapevine (10%) treatments (*p* = 0.028 and <0.001, respectively). Similarly, at Day 4, there was evidence of a difference between the Virus (85%) and both Virus/White clover (23%) and Virus/Grapevine (30%) treatments (*p* < 0.001). All first instar mealybugs sampled from negative control treatments for all blocks (No virus and No virus/White clover treatments) tested negative for GLRaV-3. GLRaV-3 was successfully detected in 17% of second instar *P. calceolariae* from the Virus treatment. One second instar *P. calceolariae*, which fed on GLRaV-3-free grapevine leaves, tested positive for GLRaV-3 (No virus, *n* = 22), suggesting contamination during the virus retention assay or total RNA extraction procedure. GLRaV-3 was not detected in second instar *P. calceolariae* from the No virus/White clover and Virus/White clover treatments. Composite exuviae samples (10 exuviae per tube) were tested for GLRaV-3. GLRaV-3 was successfully detected in 100% of exuviae samples from the Virus treatment. GLRaV-3 was not detected in exuviae from the No virus, No virus/White clover, and Virus/White clover treatments.

#### 2020 Retention Experiments: Days 1–40 on a Non-*Vitis* Host

A total of 1,288 mealybugs was tested by RT-qPCR as single mealybug samples comprising five time points; 5, 10, 16, 20, and 40 days on a non-*Vitis* host plant. Samples positive for GLRaV-3 generated Ct values that ranged between 31.5 and 35. Notably, there was a gradual reduction of mealybugs collected per time point as the experiment progressed. This result was likely because of poor mealybug settlement on the non-*Vitis* plant host (particularly for *N. benthamiana*) and the natural attrition of Crimson and White clover plants and the mealybugs they supported. The percentage of mealybugs that tested positive for GLRaV-3 by RT-qPCR reduced gradually, with no GLRaV-3 detected from 20 days on any of the non-*Vitis* host plants (Figure 1B). The percent viruliferous mealybugs on Crimson and White clover species was 19 and 18% at 5 days, 0 and 1% at 10 days, and 2 and 3% at 16 days, respectively. By contrast, at 10 and 16 days on *N. benthamiana* a greater percentage of mealybugs tested GLRaV-3 positive (7 and 31%, respectively). There was evidence of a statistically significant difference at Day 10 between Crimson clover and *N. benthamiana* (*p* < 0.01). At Day 16, there was evidence of a statistically significant difference between both clover species and *N. benthamiana* (*p* < 0.001). There was no evidence of any statistically significant difference for the *N. benthamiana* treatment between the different time points (*p* > 0.05).

### GLRaV-3 Transmission by First Instar *Pseudococcus calceolariae*

GLRaV-3 was successfully transmitted by *P. calceolariae* nymphs when they were transferred directly (Treatment 3) or indirectly (via the non-*Vitis* host White clover, Treatment 1) to recipient grapevines (Table 2). Greater transmission success was observed for direct transfer of mealybugs to the recipient grapevines compared with those that had an intermediary 5 days feeding period on the alternative host. For the first instar positive control (Treatment 3), GLRaV-3 was detected in 9 out of 10 grapevine plants based on both cane and leaf samples. By contrast, GLRaV-3 was detected in only 6 out of 10 grapevine plants based on leaf and cane samples for first instars fed intermediary on an alternate host (Treatment 1). There was no evidence of a statistically significant difference in transmission success between Treatments 1 and 3 (Fisher’s exact test; *p* = 0.23).

**TABLE 2:** Transmission of grapevine leafroll-associated virus 3 (GLRaV-3) by first and second instar *Pseudococcus calceolariae* initially fed on GLRaV-3-infected grapevine leaves and later transferred to White clover (*Trifolium repens* L., a non-*Vitis* host plant) prior to access to healthy grapevines for transmission of GLRaV-3 (Blocks 1 and 2).

**Treatment**	**AAP^*a*^**	**NVF^*b*^**	**IAP^*c*^**	**Mealybugs per plant**	**Positive plants/Inoculated plants**		**Mealybugs per plant**	**Positive plants/Inoculated plants**			
					**Block 1^*e*^**			**Block 2^*e*^**		**Combined results^*e*^**	
					**Leaf**	**Cane**		**Leaf**	**Cane**	**Leaf**	**Cane**
**1—First instar White clover**	5 days	5 days	1 week	30–37	3/5	1/5	47–52	3/5	5/5	6/10	6/10
**2—Second instar White clover**	5 days	11 days	1 week	31–38	0/4	1/4	50–52	4/5	4/5	4/9	5/9
**3—First instar No alternate host**	5 days		1 week	32–38	4/5	4/5	50	5/5	5/5	9/10	9/10
**4—Second instar No alternate host**	16 days		1 week	26–31	4/4	3/4	30–40	2/2^*d*^	3/3	6/6	6/7
**Negative controls**										0/7	0/7

*^*a*^Acquisition access period.^*b*^Non-Vitis feeding.^*c*^Inoculation access period.^*d*^Leaf samples were collected from two out of three grapevine plants, as the inoculated leaf on one of the plants senesced.^*e*^Leaf and cane material collected from same plant.*

### GLRaV-3 Transmission by Second Instar *Pseudococcus calceolariae*

Overall for Block 1 and Block 2, GLRaV-3 was successfully transmitted by second instar mealybugs when transferred directly (Treatment 4) or indirectly (via the non-*Vitis* host White clover, Treatment 2) to recipient grapevines (Table 2). Greater transmission success was observed for the second instars directly transferred (Treatment 4) compared to second instars fed on the alternate host (Treatment 2). For the second instar positive control (Treatment 4), GLRaV-3 was detected in six out of seven grapevine plants based on leaf and cane samples. In contrast when second instars were fed on White clover (Treatment 2), GLRaV-3 was detected in four out of nine recipient grapevine plants based on leaf samples, and five out of nine plants based on cane samples. There were differences in transmission success for second instars fed on White clover (Treatment 2) between Block 1 and Block 2. For Block 1, GLRaV-3 was detected in one out of four recipient grapevine plants compared to four out of five grapevine plants for Block 2. Though there was a statistically significant difference in transmission success between Block 1 and 2 for Treatment 2 (Fisher’s exact test; *p* = 0.015) there was no evidence of a difference in transmission success between Block 1 and Block 2 for Treatment 4 (*p* > 0.05). GLRaV-3 was not detected in any of the negative controls grown in parallel to the inoculated grapevines (seven plants).

### *Pseudococcus calceolariae* Internal Control

A subset of RNA extracted from mealybugs was tested for *P. calceolariae* elongation factor 1a gene and all samples were positive (data not shown).

## Discussion

### GLRaV-3 Persistence in *P. calceolariae* Nymphs: GLRaV-3 Is Retained 16 Days After Feeding on Non-*Vitis* Plant Hosts but Lost by 20 Days

This study demonstrated that more than 4 days is needed to ensure optimal virus acquisition by individual *P. calceolariae* within the mealybug populations in this study. In the 2015 retention experiment, *P. calceolariae* nymphs were given a 4 days AAP on GLRaV-3-positive grapevine leaves and showed an increased percentage of viruliferous mealybugs over time for the positive control (Virus). In the 2020 retention experiment, a 6 days AAP was used and resulted in 81% acquisition of GLRaV-3. Neither the 2015 nor the 2020 experiments resulted in a drop in GLRaV-3 Ct values over time of mealybug feeding on the non-*Vitis* host, thereby supporting a non-propagative interaction between vector and virus. Variability in virus acquisition of GLRaV-3 by mealybugs has been reported previously. Charles et al. (2006) suggested that GLRaV-3 acquisition by mealybugs is shorter than the typical AAP of other plant viruses, usually occurring within 0.25–12 h. Krüger et al. (2006) reported that *P. longispinus* first instar nymphs transmitted GLRaV-3 after an AAP of 1.5 h. Mahfoudhi et al. (2009) detected GLRaV-3 in 58.3% of first and second instar groups and 41.7% of composited adult female *P. ficus* fed on GLRaV-3 positive grapevine leaves for a 7 days AAP. Sandanayaka et al. (2013) found *P. longispinus* adults did not acquire GLRaV-3 in less than 24 h. Krüger et al. (2015) found that *P. ficus* was able to transmit GLRaV-3 after a 15-min AAP and a 15 min IAP, and for *P. longispinus*, GLRaV-3 was transmitted after an AAP of 10 min and an IAP of 1 h.

The 2015 experiment used a single set of excised GLRaV-3-positive Cabernet franc grapevine leaves on which all mealybugs fed prior to distribution to White clover or GLRaV-3-free grapevine leaves for collection at Days 1–4 (Table 1 and Figure 1). The Virus treatment comprised those mealybugs that remained on the original source leaves (Table 1). At Days 1 and 2 post initial AAP the mealybugs for the Virus treatment had only a brief time to recover from the disturbance caused by the distribution of mealybugs from those leaves. This disturbance may have resulted in less feeding and lower GLRaV-3 incidence at Days 1 and 2 compared with Days 3 and 4. Due to this presumed feeding disturbance we altered the method in the 2020 experiment to a gentler, mealybug-motivated movement from source grapevine leaves to alternative host plant leaves.

This study demonstrated that the GLRaV-3 retention period in *P. calceolariae* was at least 16 days, i.e., between when the first instar acquired the virus and subsequently fed on either White clover, Crimson clover or *N. benthamiana*. In the 2015 experiments, GLRaV-3 was detected from 23.7% of the mealybugs collected after 4 days of feeding on White clover. Furthermore, GLRaV-3 was detected from 17% of second instar citrophilus mealybugs left on GLRaV-3 positive grapevine leaves for up to 9 days then removed to Petri dishes before the mealybugs molted to become second instar mealybugs. Moreover, GLRaV-3 was transmitted to grapevines even after the inoculating mealybugs were sustained on White clover plants for 11 days. The 2020 experiments extended past 4 days access to a non-*Vitis* host and GLRaV-3 was detected in *P. calceolariae* after 16 days feeding on White clover or *N. benthamiana*, but at 20 days and beyond, no GLRaV-3 was detected in the test mealybugs.

Notably, the number of mealybugs available to be collected from *N. benthamiana* plants dramatically declined during the 2020 experiment. This result was most likely because of the poorer mealybug settlement on *N. benthamiana* compared with the clover plants. The poor mealybug settlement and consequent lack of feeding may have led to the slightly higher number of GLRaV-3 positive mealybugs from *N. benthamiana* plants compared with the clover as the virus would have had less opportunity to be transmitted from the mealybug into the solanaceous plant. GLRaV-3 detection in *N. benthamiana* plants only occurs months after inoculation (Prator et al., 2017) therefore this non-*Vitis* plant is unlikely to have provided GLRaV-3 inoculum to mealybugs within the timeframe of this retention experiment.

Reported GLRaV-3 retention time varies throughout the literature and appears to differ with mealybug species, their maturity, and the AAP. For example, it has been reported that *Planococcus citri* nymphs lose GLRaV-3 after 1 h of being removed from GLRaV-3 positive grapevines on which they had been feeding for 3 days (Cabaleiro and Segura, 1997). In another study, first and second instar nymphs of *P. longispinus* were found to retain GLRaV-3 for more than 3 days, with the percentage of viruliferous mealybugs declining over time from 81 to 17% (Krüger et al., 2006). In addition, individual *P. longispinus* first and second instar nymphs were reported to transmit GLRaV-3, after an AAP of 5 days followed by 5 days of feeding on virus-free plants (Douglas and Krüger, 2008). Krüger et al. (2015) observed GLRaV-3 retention in *P. ficus* for 8 days when feeding on a non-virus host (*Ficus benjamini*) or GLRaV-3-free grapevine, and for 2 days when starving. By contrast, *P. longispinus* retained GLRaV-3 for at least 3 days when fed on GLRaV-3-free grapevine or starving (Krüger et al., 2015). Several studies have also noted that mealybug adults (which do not feed) have lower transmission efficiency than first instar mealybugs (Petersen and Charles, 1997; Sandanayaka et al., 2013). First instar *P. longispinus* were found to start feeding earlier in their life stage and feed for longer than adults, suggesting they are more efficient vectors (Sandanayaka et al., 2013).

Collectively, based on the acquisition and retention times from previous studies, GLRaV-3 transmission by mealybugs has been described as non-circulative, non-propagative, and semi-persistent, i.e., GLRaV-3 does not breach the gut barrier of the mealybug and is retained in the stylet or foregut prior to transmission (Cabaleiro and Segura, 1997; Krüger et al., 2006, 2015; Cassone et al., 2014). Thus, GLRaV-3 is shed from the maturing mealybug with the exuviae through the molt. From the current study, the detection of GLRaV-3 in the exuviae (*n* = 6/6) and the decrease in percentage viruliferous mealybugs over time on clover or *N. benthamiana* and the eventual lack of detection of GLRaV-3 in individual mealybugs by 20 days, supports the non-circulative and non-propagative descriptors for the transmission of GLRaV-3.

If GLRaV-3 was semi-persistent, the virus would likely be bound to the stylet alimentary channel or the foregut epicuticle. Cid et al. (2007) presented results that supported circulative transmission in *Pl. citri*. When dissected after feeding on GLRaV-3 infected grapevine leaves, in all cases GLRaV-3 was detected in the salivary glands, mid-gut, hindgut, Malpighian tubes, bacteriome, and exuviae, and in some instances, in the reproductive apparatus, suboesophageal ganglion, and mouth apparatus. The virus was not detected in bundles or replication sites in *Pl. citri*, suggesting GLRaV-3 does not replicate inside the insect (Cid et al., 2007). By contrast, the dissection of *P. maritimus* after feeding on an *in vitro* solution with GLRaV-3 revealed virus accumulation in the cibarium, a pumping organ of the foregut located proximal to the esophagus (Herrbach et al., 2017), but not in other body parts (Prator and Almeida, 2020). Similarly, lettuce infectious yellows virus (LIYV; genus *Crinivirus*, family *Closteroviridae)* is transmitted by *Bemisia tabaci* (whitefly) in a non-circulative, semi-persistent transmission and LIYV was shown to localize in the anterior of the foregut or cibarium (Chen et al., 2011). The results from the current study suggests GLRaV-3 or parts of the virus are potentially moving further down the foregut of *P. calceolariae* and/or are located in parts of the insect that are not removed effectively during molt. GLRaV-3 was detected from mealybugs after an extended amount of time on a non-*Vitis* host plant (16 days) and in second instar mealybugs (*n* = 8/45), even when first instars that had been feeding on GLRaV-3-positive grapevine leaves were transferred to Petri dishes prior to molt so that mealybugs could not feed as second instars prior to virus testing. This raises the question of whether the detected GLRaV-3 is present as a whole virion and is therefore still infectious. Similar feeding/dissection studies of *P. calceolariae* will validate the GLRaV-3 binding locations for this particular mealybug species and virus, and may verify or refute the previously reported circulative manner of the GLRaV-3-vector interaction. These experiments could also be used to reveal any differences between the transmission of GLRaV-3 genetic variants (Diaz-Lara et al., 2018).

No difference in the retention of GLRaV-3 in mealybugs was observed between the two clover species that are known to be preferred hosts of *P. calceolariae* (Sandanayaka et al., 2018). Some non-*Vitis* mealybug hosts, e.g., those with high lectin content, may be capable of causing a faster decrease in mealybug GLRaV-3 retention by blocking or competing for GLRaV-3 binding to the cuticular surface of its insect vectors (Prator and Almeida, 2020). The effect of plant host, beyond grapevine, clover and *N. benthamiana*, on GLRaV-3 retention by *P. calceolariae* is yet to be determined. Given GLRaV-3 can be transmitted to at least one non-*Vitis* host i.e., *N. benthamiana*, consideration must also be given to the possibility of other plant species found within vineyards being GLRaV-3 reservoirs (Prator et al., 2017).

### GLRaV-3 Is Transmitted by Both First and Second Instar *P. calceolariae* After Feeding on Non-*Vitis* Plant Hosts of Mealybugs

The ability of second instar *P. calceolariae* to transmit GLRaV-3 was demonstrated by the 90% transmission rate by second instar nymphs following continuous feeding on GLRaV-3-infected grapevine leaves and the 40–60% transmission rate after access to white clover for up to 11 days. These data underscored the importance of mealybug control in vineyards and demonstrated that managing only the nascent mealybug instars is insufficient; long-term management is required to reduce vector-mediated GLRaV-3 transmission. A period of 16 days was assumed to be sufficiently long for the mealybugs to molt and transition into second instars. Thus, it is possible a small number of mealybugs had not molted at the time of transfer from the non-*Vitis* and *Vitis* (experimental positive control) plants. Future transmission studies will benefit from transferring mealybugs from plants to Petri dishes prior to molt, similar to the 2015 retention experiment. Furthermore, as noted above, the GLRaV-3-*P. calceolariae* relationship would benefit from investigation by feeding/dissection studies including second instar and later life stages. In particular, the use of electron microscopy to view immunologically tagged GLRaV-3 virions within mealybugs at different time points in the life-cycle and on a non-*Vitis* plant, in combination with transmission studies, would help elucidate where the infectious virion is located within the vector. The underlying question still remains, at what point in time does the GLRaV-3 virion lose its infection capacity following mealybug acquisition?

Previous studies have hypothesized that the GLRaV-3 genetic variant has an effect on the efficiency of virus transmission by mealybugs (Jooste et al., 2011; Chooi et al., 2013; Blaisdell et al., 2015). It is also possible that the genetic differences between the GLRaV-3 variants could affect the interaction between the virus and its vector, with impacts on minimal required AAP and IAP. Such differences may be direct (e.g., affecting virus retention within the insect) or indirect (e.g., affecting virus titre in the grapevine and subsequently its transmission efficiency).

### Implications for GLRaV-3 and Mealybug Management

Mealybug instars influence the spread of GLRaV-3 within and between vineyards by crawling between vines, movement via vineyard management activities and aerial dispersal (Charles et al., 2009). Dispersal of viruliferous mealybug nymphs result in vine-to-vine spread of GLRaV-3, which is typically most pronounced within vine rows (Bell et al., 2018). Several epidemiology studies supported this form of dispersal, with infection shown to spread more rapidly along rows than between rows and often appeared clustered (Cabaleiro and Segura, 1997). Vine management activities such as machinery use, leaf trimmers, and machine harvesters may also transport mealybugs within the vineyard and between adjoining vineyards (Charles et al., 2009). Aerial dispersal of mealybugs, particularly nymphs can result in movement of GLRaV-3 between blocks and vineyards (Charles et al., 2006). There is a strong correlation between mealybug numbers and GLRaV-3 infection levels in the subsequent seasons; accordingly the rapid spread of GLRaV-3 is purported to be a consequence of high-density mealybug populations (Charles et al., 2009; Franco et al., 2009; Daane et al., 2012).

Here, we have shown that GLRaV-3 can be retained and transmitted by first or second instar *P. calceolariae* after either feeding on a non-*Vitis* mealybug host or GLRaV-3-free grapevine leaves for up to 16 days. These results demonstrated the insidious nature of GLRaV-3 in vineyards and they go some way to explaining issues around virus persistence, spread, and barriers to effective management. Measures to control GLRaV-3 spread that do not rely on insecticides only could include the use of ground-cover plants that host the mealybug vector but not the virus, thereby interrupting the GLRaV-3 transmission pathway from grapevine to grapevine. However, alternative hosts like clover must be a sink for mealybugs for a period of time sufficient to transition viruliferous individuals to non-viruliferous. In New Zealand, this novel addition to the integrated response to GLRaV-3 management is being evaluated (V. Bell unpublished data), and comes at a time when the wine sector is actively reducing its reliance on insecticides.

## Data Availability Statement

The raw data supporting the conclusions of this article will be made available by the authors, without undue reservation.

## Author Contributions

MS, KC, and RM: conceptualization and supervision. BM, RG, RR, KC, VD, and CM: data generation and curation. BM, KC, RG, and KR: formal analysis. KC, RM, and VB: Funding acquisition. BM, MS, RG, RR, VD, CM, KR, VB, KC, and RM: methodology, writing—review, and editing. BM, MS, KC, RM, and RG: writing—original draft. All authors contributed to the article and approved the submitted version.

## Conflict of Interest

The authors declare that the research was conducted in the absence of any commercial or financial relationships that could be construed as a potential conflict of interest.
